# Case report: Rapid development of amyloid A amyloidosis in temporal arteritis with *SAA1.3* allele; An unusual case of intestinal amyloidosis secondary to temporal arteritis

**DOI:** 10.3389/fimmu.2023.1144397

**Published:** 2023-03-21

**Authors:** Shuhei Yoshida, Haruki Matsumoto, Jumpei Temmoku, Norshalena Shakespear, Yuichiro Kiko, Kentaro Kikuchi, Yuya Sumichika, Kenji Saito, Yuya Fujita, Naoki Matsuoka, Tomoyuki Asano, Shuzo Sato, Eiji Suzuki, Hiroshi Watanabe, Hiromasa Ohira, Kiyoshi Migita

**Affiliations:** ^1^ Department of Rheumatology, Fukushima Medical University School of Medicine, Fukushima, Japan; ^2^ Department of Diagnostic Pathology, Fukushima Medical University School of Medicine, Fukushima, Japan; ^3^ Department of Gastroenterology, Fukushima Medical University School of Medicine, Fukushima, Japan; ^4^ Department of Rheumatology, Ohta-Nishinouchi Hospital, Koriyama, Japan

**Keywords:** amyloid A amyloidosis, tocilizumab, large-vessel vasculitis, serum amyloid A, *SAA1* polymorphism, temporal arthritis

## Abstract

Temporal arteritis (TA) is a large-vessel vasculitis mostly seen in older patients. Amyloid A (AA) amyloidosis secondary to a chronic inflammation induces multiple organ dysfunctions, including a dysfunction of the gastrointestinal tract. Herein, we present a case of TA complicated by AA amyloidosis that was resistant to oral and intravenous steroids. An 80-year-old man with a history of new-onset headache, jaw claudication, and distended temporal arteries was referred to our department. On admission, the patient presented with tenderness and a subcutaneous temporal nodule in both temple arteries. Ultrasonography of the nodule revealed an anechoic perivascular halo surrounding the right temporal artery. Following the diagnosis of TA, high-dose prednisolone therapy was initiated. However, the patient presented with recurrent abdominal pain and refractory diarrhea. Due to the unclear origin of refractory diarrhea, an extensive workup, including biopsy of the duodenal mucosa, was performed. Endoscopy revealed chronic inflammation in the duodenum. Immunohistochemical analysis of duodenal mucosal biopsy samples revealed AA amyloid deposition resulting in the diagnosis of AA amyloidosis. After tocilizumab (TCZ) administration, refractory diarrhea reduced; however, the patient died of intestinal perforation 1 month after the start of TCZ administration. Gastrointestinal involvement was the main clinical manifestation of AA amyloidosis in the present case. This case highlights the importance of bowel biopsy screening for amyloid deposition in patients with unexplained gastrointestinal tract symptoms, even in a recent onset of large-vessel vasculitis. In the present case, the carriage of the *SAA1.3* allele likely contributed to the rare association of AA amyloidosis with TA.

## 1 Introduction

Temporal arteritis (TA) is a systemic vasculitis that affects large- and medium-sized arteries, and it is commonly seen in older patients (1). Amyloidosis is characterized by local or systemic deposition of insoluble protein aggregates in organs (2). Amyloid A (AA) [secondary] amyloidosis is characterized by the extracellular deposition of misfolded AA amyloid fibrils, which are proteolytic cleavage products of serum AA (SAA) protein (3). SAA is mainly synthesized by hepatocytes as an acute-phase reactant *via* the stimulation of inflammatory cytokines, including interleukin-6 (4). AA amyloidosis occurs in patients with uncontrolled rheumatic diseases with inflammation (5). The most frequent diseases that underlie AA amyloidosis are rheumatoid arthritis (RA) and autoinflammatory diseases (6). AA amyloidosis secondary to TA is extremely rare (7). It has been speculated that in addition to the inflammatory processes of rheumatic diseases, genetic factors may contribute to the development of AA amyloidosis (8, 9). For example, AA amyloidosis has been previously described in patients with Familial Mediterranean fever, such as homozygosity for the pyrin mutation p.M694V (10).

In AA (secondary) amyloidosis, protein fibrils are composed of proteins fragments of SAA (2). The presence of two single-nucleotide polymorphisms (SNPs) within exon 3 of *SAA1* gene, 2995C/T and 3010C/T, define three haplotypes that correspond to *SAA1.1*, *SAA1.3*, and *SAA1.5* (8). The development of AA amyloidosis has been demonstrated to be positively related to the frequency of *SAA1.3* alleles in the Japanese population (11, 12). We describe an older male patient whose clinical manifestations were consistent with TA symptoms, including bitemporal headaches with localized tenderness on palpation and jaw claudication, and elevated inflammatory markers. Our patient rapidly developed AA amyloidosis from the first clinical manifestation of TA. However, it is unusual for AA amyloidosis to manifest almost simultaneously with the initial symptoms of primary diseases. Herein, we discuss this rare association and highlight the mechanism of rapidly developing AA amyloidosis in rheumatic disorders.

## 2 Case presentation

**Figure 1:**
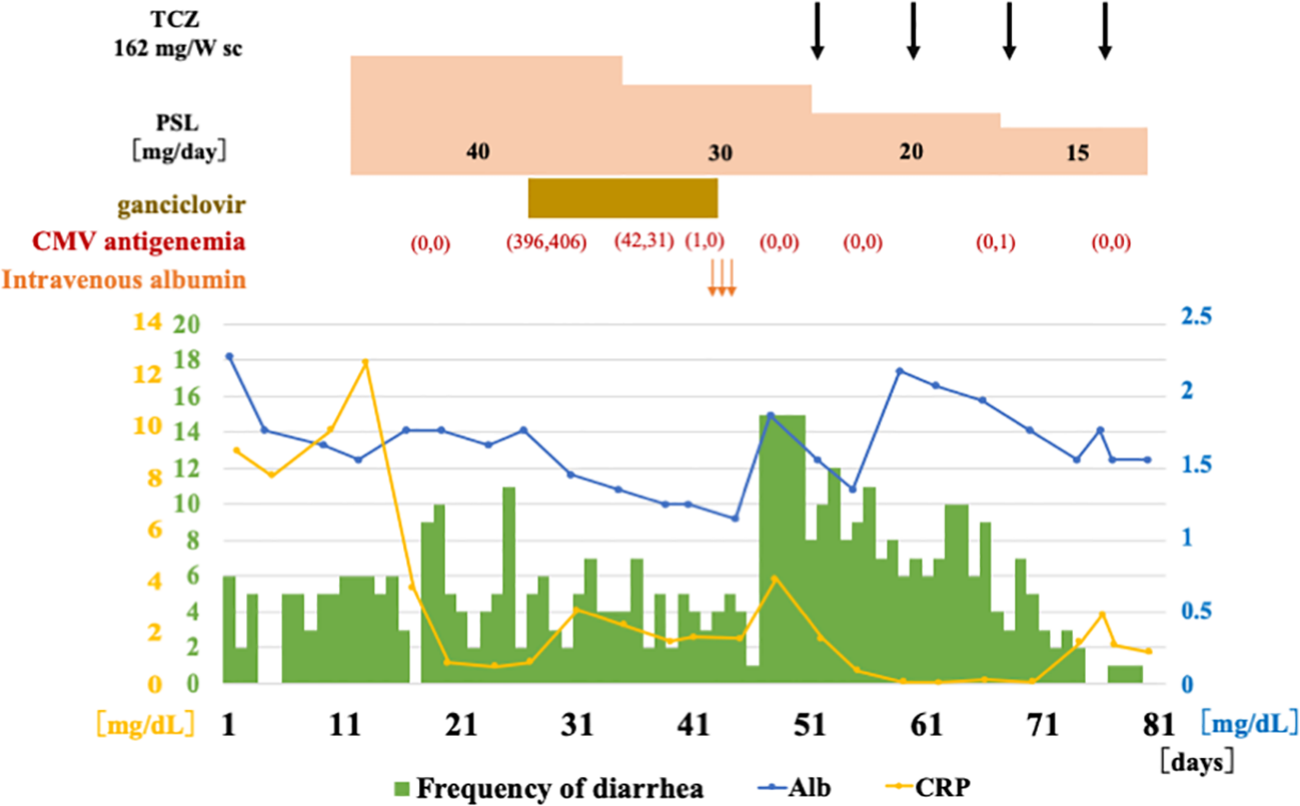
Clinical course after admission. After the diagnosis of giant cell arteritis, prednisolone 40 mg (0.6 mg/kg body weight) was administered orally. During the course, CMV-infected cells became positive, and ganciclovir was administered. After the diagnosis of AA amyloidosis, subcutaneous injections of TCZ was initiated. Diarrhea tended to improve and serum albumin tended to increase. Glucocorticoid therapy was tapered off, but the patient died and was discharged on the 80th day due to sigmoid colon perforation. TCZ, tocilizumab; sc, subcutaneous injection; PSL, prednisolone; CMV, cytomegalovirus; Alb, albumin; CRP, C-reactive protein.

**Figure 2:**
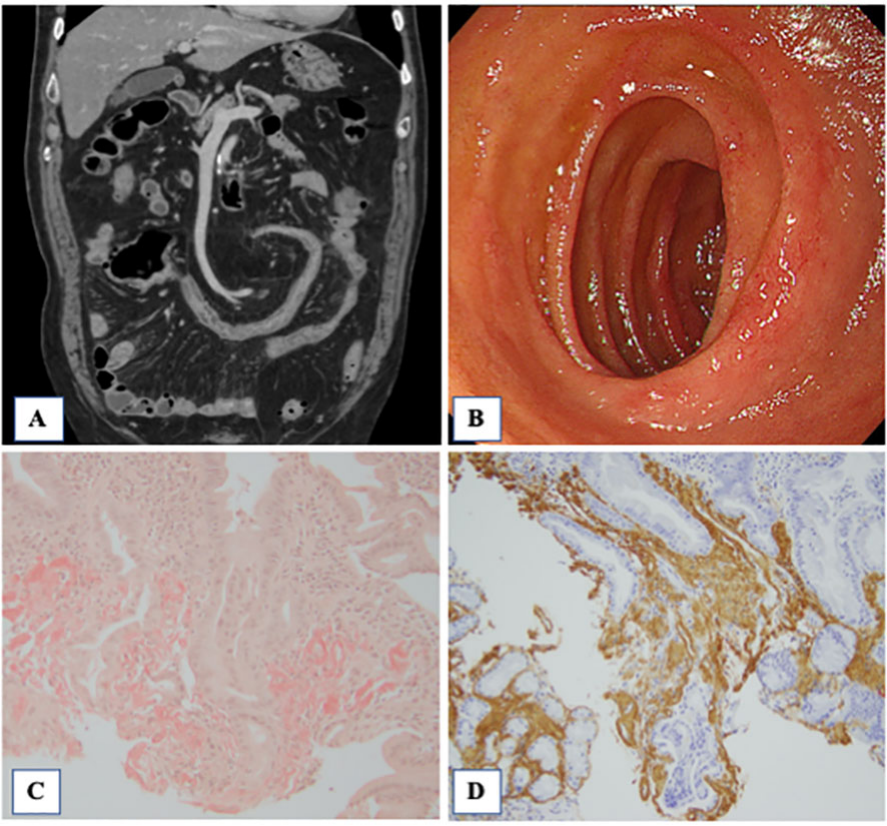
Contrast-enhanced CT, endoscopy and pathological findings of the biopsied intestinal mucosa. **(A)** Contrast-enhanced CT showed wall thickening in the small intestine and colon. In addition, there were multiple diverticula in the colon. **(B)** Redness of the mucosa of the duodenum was observed. Histopathological findings of the duodenal mucosa. the Congo red method was used to detect amyloid in tissue sections. Amyloid was identified as the AA type on immunostaining using monoclonal antibodies specific to amyloid A (AA). **(C)** The amorphous extra-cellular material in the intrinsic layer of the duodenal mucosa was positively stained with Congo red (×100, Congo red). **(D)** Extra-cellular and peri-vascular deposits of amyloid reveal positive immunoreactivity with an antibody against amyloid A (×100).

An 80-year-old man was admitted to a local hospital with fever, headache, thigh pain, and frequent diarrhea. The patient underwent artificial vessel replacement for a ruptured abdominal aortic aneurysm at the age of 69 years and laparoscopic cholecystectomy for acute cholecystitis at the age of 76 years. The patient had no history of autoinflammatory or autoimmune diseases. The patient had a 51-year history of smoking 30 cigarettes a day since the age of 18. The patient had been asymptomatic for more than 10 years while regularly attending hospital since the abdominal aortic replacement surgery, and blood tests showed normal serum C-reactive protein (CRP) levels. Four months prior to admission, the patient experienced fatigue, but blood tests at that time revealed normal serum CRP levels (0.13 mg/dL; normal range [NR]: up to 0.30 mg/dL) and no evidence of increased inflammation. One month prior to admission, low-grade fever approximately 37°C and headache appeared. Two weeks prior to admission, the patient presented with morning stiffness, general malaise, and five to six episodes of soluble diarrhea in one day, which did not improve; therefore, the patient visited his previous physician. Blood tests performed by the previous physician revealed elevated serum CRP levels (7.89 mg/dL; NR: up to 0.30 mg/dL). Computed tomography (CT) revealed no abnormalities indicating the cause of the fever. Blood and stool cultures did not isolate any causative organisms. Because infection of the artificial blood vessel could not be ruled out, the patient was treated with antimicrobial agents. However, antimicrobial treatment did not resolve the fever. Temporal artery ultrasonography performed to investigate the headache revealed circumferential wall thickening of the right shallow temporal artery, which led to suspicion of giant cell arteritis. The patient was later transferred to our department for treatment. Physical examination revealed tenderness in both temporal arteries, flat and soft abdomen, and slightly increased intestinal peristalsis. Laboratory examination data are shown in **Table 1**. Acute phase reactants were elevated, and myeloperoxidase-anti-neutrophil cytoplasmic antibodies (MPO-ANCA) were positive, with a low titer. Electrocardiography and echocardiography revealed no abnormal findings. Contrast-enhanced MRI revealed no evidence of intracranial vascular lesions. A repeat contrast-enhanced computed tomography (CT) scan showed mild wall thickening of the entire small intestine, although there were no findings suggestive of a malignancy. The patient had symptoms of proximal myalgia and morning stiffness similar to polymyalgia rheumatica. In addition, the patient met the 2022 American College of Rheumatology (ACR)/EULAR classification criteria for giant cell arteritis (13) with a score of 12: age ≥50 years at the time of diagnosis, halo sign on temporal artery ultrasound, maximum CRP ≥10 mg/dL, morning stiffness in the shoulders, and new temporal headache. Although the patient was positive for MPO-ANCA, the clinical course and imaging findings ruled out microscopic polyangiitis. The patient was diagnosed with giant cell arteritis and polymyalgia rheumatica. The clinical course of the patient is summarized in **Figure 1**. On the day 12, treatment was initiated with oral prednisolone 40 mg/day (0.6 mg/kg body weight). The fever and myalgia quickly improved, and inflammatory marker levels decreased. However, anorexia and diarrhea persisted. Because of progressive hypoalbuminemia, central venous nutrition was initiated and prednisolone was changed to intravenous administration. Subsequently, thrombocytopenia was confirmed, CMV infection was suspected, and positive cells were detected in a repeated CMV antigenemia test. Therefore, considering the possibility of diarrhea due to CMV enteritis, intravenous ganciclovir was administered. After starting ganciclovir, CMV antigenemia was eliminated. Intravenous ganciclovir was continued for 3 weeks without improvement in diarrhea. A repeated contrast-enhanced CT showed wall thickening of the small intestine and colon (**Figure 2**). Lower gastrointestinal endoscopy showed residual feces in the colon due to inadequate pretreatment, and although erosions and erythema of the mucosa were observed, they could not be adequately observed. Upper gastrointestinal endoscopy revealed edematous duodenal mucosa (**Figure 2**). A biopsy was performed at the same site. Histopathology revealed amyloid deposits in the intrinsic layer of the duodenal mucosa by Congo red staining (**Figure 2**), and AA amyloidosis was diagnosed by immunostaining (**Figure 2**). As prednisolone alone did not improve diarrhea caused by AA amyloidosis, tocilizumab 162 mg/week was started on day 48. The diarrhea improved gradually; however, on day 71, the patient developed somnolence and complained of mild abdominal pain. Therefore, a contrast-enhanced CT scan was performed the next day, and the scan revealed massive ascites accumulation and perforation of the sigmoid colon. The patient was immediately referred to the gastrointestinal surgery department; however, surgery was not feasible because of the patient’s immunosuppressed state, poor general condition, and large amount of ascites. Conservative treatment with antimicrobial agents was administered. On day 73, the patient developed dyspnea and was started on oxygen therapy. After consultation with the family, the patient was placed on palliative care and started on continuous intravenous morphine hydrochloride. The patient’s blood pressure gradually decreased, and the patient died on day 80.

**Table 1:** Laboratory findings on admission.

Peripheral blood		Serological tests	
Red blood cells	4.76 × 10^6/^μL	C-reactive protein	8.28 mg/dL (<0.30)
Hemoglobin	13.4 g/dL	Ferritin	498 ng/mL (50–200)
Hematocrit	41.4%	KL-6	476 U/mL (<499)
Platelet	289 × 10^3/^μL	SAA	98.7 mg/L (<3.0)
White blood cells	8,700/μL	IgG	1811 mg/dL (861–1747)
Neutrophil	73.0%	IgA	273 mg/dL (93–393)
Eosinophil	8.0%	IgM	34 mg/dL (33–183)
Monocyte	6.0%	C3	166 mg/dL (73–138)
Lymphocyte	13.0%	C4	34 mg/dL (11–31)
Basophil	0.0%	ANA	<160 (1:160)
**Blood chemistry**		Anti-ds-DNA Abs	0.7 U/mL (<9.9)
Total protein	7.2 g/dL (6.6–8.1)	Anti-Sm Abs	1.0 U/mL (<6.9)
Total bilirubin	0.6 mg/dL (0.4–1.5)	Anti-RNP Abs	1.0 U/mL (< 4.9)
Albumin	3.0 g/dL (4.1–5.1)	Anti-SS-A Abs	33 U/mL (<6.9)
Aspartate aminotransferase	20 IU/L (13–30)	Anti-SS-B Abs	0.7 U/mL (<6.9)
Alanine aminotransferase	14 IU/L (10–42)	Anti-CCP Abs	<0.6 U/mL (<4.5)
Lactate dehydrogenase	184 IU/L (124–222)	Rheumatoid factor	<5 IU/mL (<15)
Γ-Glutamyl transpeptidase	20 IU/L (13–64)	MPO-ANCA	21 EU (0-3.5)
Alkaline phosphatase	78 IU/L (38–113)	PR3-ANCA	< 0.6 EU (0-2)
Creatine kinase	42 U/L (59–248)	Anti-Cardiolipin Abs	1.3 U/mL (0–9.9)
Blood urea nitrogen	19 mg/dL (8–20)	Lupus anticoagulant	1.1 (<1.3)
Creatinine	1.13 mg/dL (0.65–1.07)	Anti-GBM Abs	1.7 U/mL (0–7.0)
Na	134 mEq/L (138–145)	Anti-centromere Abs	< 5.0 U/mL (<10)
K	3.9 mEq/L (3.6–4.8)	**Urinalysis**	
Cl	99 mEq/L (101–108)	pH	5.5
Glucose	121 mg/dL (73–109)	Specific gravity	1.018
Hemoglobin A1c	7.6% (4.9–6.0)	Protein	(±) 0.34 g/g Cre
TSH	1.061 μIU/mL (0.5–5)	Occult blood	(-)
Free T3	2.78 pg/mL (2.3–4)	Bacteria	(-)
Free T4	1.14 ng/dL (0.9–1.7)	Red blood cells	0-1/HPF
**Coagulation tests**		White blood cells	0-1/HPF
Prothrombin time	78.6% (70-130)	Hyaline casts	100-999/WF
activated partial thromboplastin time	28.8 seconds (26.9-38.1)	Granule casts	50-99/WF
		**Microbiologocal tests**	
		HBs Ag	(-)
		Anti-HCV Abs	(-)
		Nontreponemal test	(-)

TSH, thyroid stimulating hormone; SAA, serum amyloid A; KL-6, Sialylated carbohydrate antigen KL-6; Ig, immunoglobulin; ANA, antinuclear antibodies; Abs, antibodies; Anti-ds-DNA, anti-double stranded-DNA; Anti-Sm, anti-smith; Anti-RNP, anti-ribonucleoprotein; Anti-CCP, anti-cyclic citrullinated peptide; MPO-ANCA, myeloperoxidase-anti-neutrophil cytoplasmic antibodies; PR3-ANCA, proteinase 3-anti-neutrophil cytoplasmic antibodies; Anti-GBM, anti-glomerular basement membrane; Cre, creatine; HPF, High power field; WF, whole field; HBsAg, hepatitis B virus surface antigen; Anti-HCV Abs, anti-hepatitis C virus antibodys.

### 2.1 Analysis of SAA1 gene genotyping

The *SAA1.1*, *1.3*, and *1.5* alleles, corresponding to the T-C, C-T, and C-C haplotypes of the C2995T (rs1136743) and C3010T (rs1136747) polymorphisms, were also determined using the PCR- restriction fragment length polymorphism method (14). The primers used for the PCR reaction were 5′-GCCAATTACATCGGCTCAG-3′ (sense) and 5′-TGGCCAAAGAATCTCTGGAT-3′ (antisense). The 518-bp PCR products were digested with restriction enzyme BclI (Promega, San Luis Obispo, CA, USA) and BanI (Promega) and electrophoresed on a 2.5% agarose gel (14).

**Figure 3:**
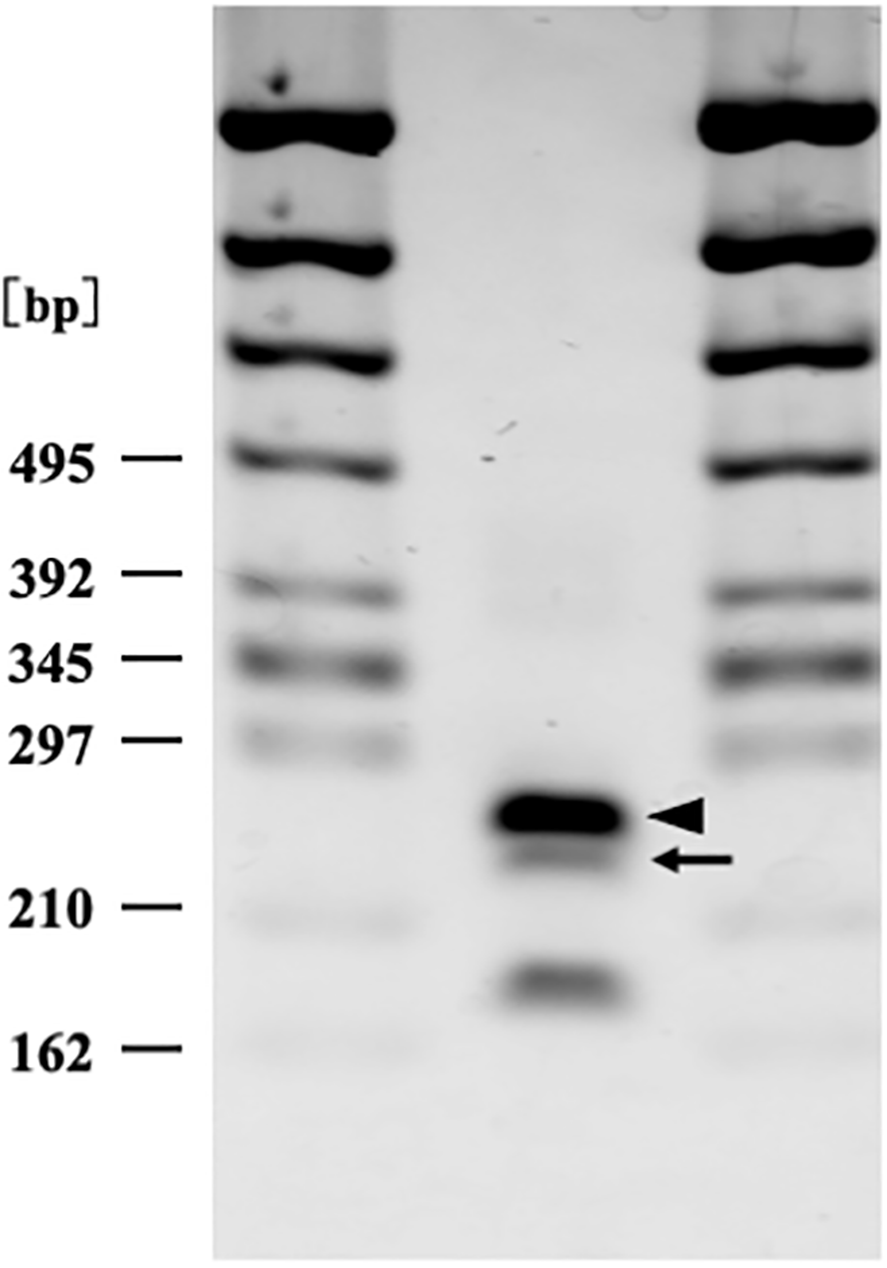
Serum amyloid A1 genotyping by polymerase chain reaction restriction fragment length polymorphism (PCR-RFLP) analysis (2% agarose gel electrophoresis). Arrow (250 bp) and arrow head (269 bp) correspond to serum amyloid A1 allele 1.5 and 1.3 respectively. The patient carried SAA 1.3/1.5 heterozygosity genotype.

The results showed that the patient carried *SAA1.3*/*SAA1.5* genotype (**Figure 3**).

## 3 Discussion

AA amyloidosis is a serious complication associated with various inflammatory disorders (15). Vasculitis, an autoimmune chronic inflammatory disease, is characterized by elevated levels of acute-phase proteins, including SAA and CRP, similar to other inflammatory disorders (16). We described an elderly patient with TA who presented with refractory diarrhea secondary to AA amyloidosis almost simultaneously with the onset of TA. In the present case, persistent vascular inflammations caused by TA for 3 months was regarded as the primary inflammatory focus for AA amyloidosis. After intensive workup, immunohistochemical analysis showed submucosal AA amyloid deposits in the duodenum, which was also verified by high levels of circulating SSA (98.7 mg/dl; normal range: up to 3.0 mg/dL). We concluded that refractory diarrhea was caused by intestinal AA amyloidosis secondary to TA. The most frequent clinical manifestations of AA amyloidosis are renal dysfunction and proteinuria (15). However, AA amyloidosis also induces gastrointestinal involvement, including refractory diarrhea, malabsorption and paralytic ileus (15). Thus, it is important to perform an intestinal workup, including the screening for intestinal AA amyloidosis, in individuals with various types of malabsorption syndromes or refractory gastrointestinal disorders complicated by rheumatic disorders (17).

The severity and durations of the primary inflammatory diseases may contribute to the development of AA amyloidosis (5). The durations between clinical manifestations of AA amyloidosis and the underlying inflammatory diseases can vary (6). Studies investigating AA amyloidosis in RA patients reported that the period of latency between the onset of RA and AA amyloidosis are17–26 years (18). We presented a case of AA amyloidosis which almost simultaneously complicated with new-onset TA. It can be speculated that in addition to the inflammatory processes of TA, genetic factors may contribute to the rapid development of AA amyloidosis. SAA1 is the major constituent of AA protein (19). *SAA1* gene polymorphisms affect both SAA transcriptional activity and the differences in enzymatic SAA1 proteolysis, suggesting the disease-modifying effects of the *SAA1* genotype (20). In Japanese patients, the *SAA1.3* allele is a high-risk factor for AA amyloidosis (11). Furthermore, the *SAA1.3*/*1.3* genotype in Japanese patients with RA was associated with a shorter latency before the onset of AA amyloidosis and more severe AA amyloidosis-related symptoms (21). *SAA1.3* risk allele in the *SAA1* gene demonstrated in the present case may predispose to AA amyloidosis during the limited periods of TA-related inflammation. In the present case, elderly age, in addition to genetic factors, could be linked with AA amyloidosis through the exacerbation of chronic inflammation in the present case. Individuals over 70 years of age are susceptible to developing AA amyloidosis after chronic inflammation lasting for a relatively short period (just a few years) (22).

The resolution of AA amyloid deposits appears to begin when the inflammation subsides with normalized SAA levels (6). Conventional treatment strategies for AA amyloidosis mostly depend on the control of the underlying disorder as well as the normalization of circulating SAA (5, 23). A central role for interleukin 6 (IL-6) in the pathogenesis of AA amyloidosis has been suggested, because IL-6 is a critical inducer of SAA (24, 25). IL-6 inhibition may result in the suppression of SAA levels, improvement of the clinical symptoms of AA amyloidosis, and regression of intestinal AA fibril deposition. Therefore, treatment with IL-6 receptor antagonists is a potential therapeutic strategy for AA amyloidosis. In patients with AA amyloidosis, normalization of SAA levels is the major treatment goal, resulting in the resolution of AA amyloidosis-related manifestations. The ability of tocilizumab to improve AA amyloidosis complicated with RA has already been reported in a case-control study (26). The effectiveness of TCZ has been demonstrated in patients with end-stage renal failure and underlying RA (27).

The mainstay of TA treatment is glucocorticoid therapy (28). However, the Giant-Cell Arteritis Actemra (GiACTA) trial showed that tocilizumab (TCZ) greatly increased the rate of sustained remission of giant cell arteritis (29). Based on the results of randomized controlled trials, TCZ has been approved for the treatment of GCA. Therefore, the therapeutic use of TCZ is reasonable in patients with GCA who fail to respond to other treatments including steroid. However, TCZ is associated with an unexplained increased rate of bowel perforation (30). The British Society for Rheumatology guidelines (31) recommend the use of TCZ with caution in patients with a history of diverticular disease. In addition, it is possible that tocilizumab merely promotes the development of colon perforation while dampening the accompanying inflammatory markers *via* IL-6 inhibition. The exact mechanism of bowel perforation is unclear and is likely multifactorial in the present case. A previous study on patients receiving TCZ showed that the overall risks of diverticulitis or lower gastrointestinal perforations were low; however, the risks were higher than those of other biological disease modifying anti-rheumatic drugs (DMARDs) (32). Further studies are required to evaluate whether the increased risk of GI perforation is specific to TCZ. Previous case reports have concluded that TCZ could improve severe life-threatening diarrhea associated with AA amyloidosis secondary to RA (33). However, rheumatologists should be aware of this potentially life-threatening complication in patients with amyloid-related gastrointestinal complications, and the risk of intestinal perforation should be carefully assessed before the initiation of TCZ therapy.

In conclusion, we described a TA patient with refractory diarrhea caused by massive deposition of AA protein in the gastrointestinal tract. The present case suggests that refractory diarrhea can be complicated by TA as a consequence of AA amyloidosis. Although the underlying mechanisms for the rapid occurrence of this rare association between TA and amyloidosis remain unclear, carriage of the *SAA1.3* allele is likely to be linked to poor outcomes. AA amyloidosis remains a life-threatening disease, with an unmet need for prevention and effective treatment, even in large-vessel vasculitis.

## Data availability statement

The datasets presented in this study can be found in online repositories. The names of the repository/repositories and accession number(s) can be found in the article/supplementary material.

## Ethics statement

Ethical review and approval was not required for the study on human participants in accordance with the local legislation and institutional requirements. The patients/participants provided their written informed consent to participate in this study. Written informed consent was obtained from the next of kin for the publication of any potentially identifiable images or data included in this article.

## Author contributions

SY, ES, and KM were involved with the conception of the work. HM, JT, NS, YK, KK, YS, KS, YF, NM, TA, SS, HW, and HO contributed to the treatment and collection of data. NS and YK performed histopathological evaluation of the duodenal mucosa. SY and KM wrote the first draft of the manuscript. All authors contributed to the article and approved the submitted version.
